# Transcriptomic Analysis Reveals *CBF*-Dependent and *CBF*-Independent Pathways under Low-Temperature Stress in Teak (*Tectona grandis*)

**DOI:** 10.3390/genes14112098

**Published:** 2023-11-18

**Authors:** Miaomiao Liu, Guang Yang, Wenlong Zhou, Xianbang Wang, Qiang Han, Jiange Wang, Guihua Huang

**Affiliations:** 1 Research Institute of Tropical Forestry, Chinese Academy of Forestry, Guangzhou 510520, China; lmm19971220@stu.jxau.edu.cn (M.L.); ritfkyc@caf.ac.cn (G.Y.); zhouwl2000@caf.ac.cn (W.Z.); wangxb@caf.ac.cn (X.W.); hanqiang1988@caf.ac.cn (Q.H.); 2 College of Forestry, Jiangxi Agricultural University, Nanchang 330045, China

**Keywords:** teak (*Tectona grandis*), low temperature stress, transcriptome, *CBF*-dependent pathway, *CBF*-independent pathway

## Abstract

Teak is a rare tropical tree with high economic value, and it is one of the world’s main afforestation trees. Low temperature is the main problem for introducing and planting this species in subtropical or temperate zones. Low-temperature acclimation can enhance the resistance of teak to low-temperature stress, but the mechanism for this is still unclear. We studied the gene expression of two-year-old teak seedlings under a rapid temperature drop from 20 °C to 4 °C using RNA-seq and WGCNA analyses. The leaves in the upper part of the plants developed chlorosis 3 h after the quick transition, and the grades of chlorosis were increased after 9 h, with the addition of water stains and necrotic spots. Meanwhile, the SOD and proline contents in teak leaves increased with the prolonged cold stress time. We also identified 36,901 differentially expressed genes, among which 1055 were novel. Notably, *CBF2* and *CBF4* were significantly induced by low temperatures, while *CBF1* and *CBF3* were not. Furthermore, WGCNA successfully identified a total of fourteen modules, which consist of three modules associated with cold stress response genes, two modules linked to *CBF2* and *CBF4*, and one module correlated with the *CBF*-independent pathway gene *HY5*. The transformation experiments showed that *TgCBF2* and *TgCBF4* improved cold resistance in *Arabidopsis* plants.

## 1. Introduction

*Tectona grandis*, commonly known as teak, is a tree species in the Verbenaceae family that produces one of the finest hardwoods globally. The high demand for this hardwood has caused log prices to range from $ 600/m^3^ to 1000/m^3^ [1], and its cultivation area has expanded to over 50 countries and regions. Expanding the planting of teak will play a crucial role in the future because of the excessive felling of teak natural forests and increasing demand. However, teak, a wood commonly found in tropical regions, has encountered difficulties due to low temperatures in suboptimal areas.

Temperature is a critical element that significantly impacts the growth and distribution of plants [2]. Mild low-temperature stress triggers reversible changes in plants’ morphology, structure, physiology, and metabolism. On the other hand, severe stress causes irreversible changes that can result in plant death [3]. Due to their immobility, plants have developed various survival strategies for enduring low-temperature stress. When subjected to low-temperature stress, genes associated with osmotic adjustment, photosynthesis, antioxidation, and detoxification exhibit molecular-level responses, facilitating plant survival through the protection and restoration of cellular structure and function, as well as the induction of changes across various levels, from molecules to individuals [4]. Furthermore, following cold acclimation, plants significantly increase their resistance to low temperatures.

The transmission of signals triggered by cold stress is characterized by the participation of numerous gene responses, factors, and hormones, forming a complex network of interactions. The *ICE*-*CBF*-*COR* pathway has been extensively studied among these pathways [5]. The core gene in this pathway, *CBFs* (C-repeat binding factor), is induced by *ICEs* (inducer of *CBF* expression) to activate *CORs* (cold-responsive). Generally, in *Arabidopsis*, cold induces three *CBFs* (*CBF1*, *CBF2*, *CBF3*), while drought and salt stress activate *CBF4* [6]. Additionally, the *ICE* possesses a MYC structure and functions as a basic helix–loop–helix (bHLH) transcription factor. Although *ICE1* expression is constitutive, it rarely influences *CBF3* expression [7]. However, it can enhance *CBF3* expression under low-temperature conditions. Also, *ICE2* and *ICE1* show a high degree of homology and are involved in regulating *CBF1* [8]. Generally, *CBF1* and *CBF3* take precedence over *CBF2* in terms of expression, and *CBF2* acts as a negative regulator for *CBF1* and *CBF3* during low-temperature stress. *CBF1* has a limited impact on *CBF3* expression, and there are a few feedback loops involving *CBF1*, *CBF3*, and *CBF2* [9]. The *CBF* activates *COR* expression by binding to the cis-acting element DRE/CRT in the promoter, which has a core sequence ‘CCGAC.’ Two or three *CBFs* jointly regulate over two-thirds of *CORs*, while a single *CBF* controls the remaining *CORs* [10].

Additionally, several regulatory factors located upstream of *CBFs* are involved in regulating the *ICE*-*CBF*-*COR* pathway. One such factor is *CAMTA*, which positively influences the expression of *CBF1* and *CBF2* [11]. For instance, the expression of *CBF1-3* can be regulated by *CAMTA1* and *CAMTA2*, while *CAMTA3* mediates *CBF2* [12,13]. Additionally, the regulation of *CBF1* expression by *CAMTA3* and *CAMTA5* is commonly seen during a rapid temperature drop, but their role is seldom observed during a gradual decrease [11]. In addition, *MYB15* functions as a repressor of this pathway, preventing *CBF* expression. Surprisingly, *ICE1* can also decrease *MYB15* expression. Furthermore, *ICE1* is degraded by the ubiquitin ligase HOS1 and the SUMO ligase SIZ1, resulting in its stability and limited degradation [14,15].

There are two ways in which plant hormones are involved in the response to cold stress. ABA (abscisic acid), JA (jasmonic acid), SA (salicylic acid), BR (brassinosteroid), and ET (ethylene) are involved in the plant response to cold stress by impacting CBF either individually or in combination. In contrast, the effects of Auxin and CK (cytokinin) are attributed to the modulation of hormone transporters under cold stress conditions, consequently impacting concentration gradients [16,17]. Moreover, downstream gene activation can be directly triggered by *HY5* (enhanced hypocotyl 5), *REV4* (reville4), *REV8* (reville8), and *CCA1* (circular clock associated 1) without the involvement of the *CBF* pathway [18,19].

Despite the importance of *ICE*-*CBF*-*COR* as a response pathway to cold stress, it is worth noting that *CBFs* only govern a mere 10–20% of *CORs*, and even triple mutants of *CBFs* still retain the ability to undergo cold acclimation [9]. In regions with temperate climates, plants can adapt to cold stress by regulating gene expression and metabolism at non-freezing temperatures, resulting in improved cold resistance. However, tropical plants rarely experience low temperatures throughout their lifespan, making them vulnerable to such conditions. The teak tree is a species that is typically found in tropical climates. It requires temperatures between 25 and 30 °C for optimal growth. It should be noted that they are prone to injury at temperatures of 15 °C and below. Seedlings and young forests can experience mortality due to short-term exposure to temperatures below 4.5 °C [20].

To comprehend the cold resistance mechanism of teak during rapid temperature drops, we employed RNA-seq and WGCNA methodologies to assess gene expression alterations in two-year-old teak seedlings at three distinct time intervals after a sudden temperature shift from 20 °C to 4 °C. Furthermore, we analyzed the hub gene and its corresponding regulatory network. The results offer data for studying the cold resistance mechanism of teak and serve as a reference for genetic breeding.

## 2. Materials and Methods

### 2.1. Materials and Treatments

Two-year-old teak variety 7514 was planted in a nursery located at the Institute of Tropical Forestry, Chinese Academy of Forestry in Guangzhou (Guangzhou, N 113°21′40″, E 23°7′28″). In late November 2015, the seedlings of teaks were transplanted into a greenhouse for treatment preparation. The conditions of the greenhouse were set at day/night temperatures of 20 °C/18 °C, a photoperiod of 16/8 h, and a light intensity of 90 μmol·m^−2^·s^−1^. After 14 days of transplanting, the plants were treated at 4 °C and then sampled at 0 h, 3 h, and 9 h, respectively. Each sample consisted of the functional leaves of at least three seedlings or more, and there were three biological repetitions. The samples were frozen with liquid nitrogen and stored in a −80 °C refrigerator. Next, the samples were used for the determination of the physiological index, transcriptomic library construction, and real-time quantitative PCR (qRT-PCR) validation.

### 2.2. Determination of Free Proline and Superoxide Dismutase

The free proline content was determined by sulfosalicylic acid-acidic ninhydrin, and the colorimetry was set at 520 nm. Additionally, the activity of SOD was measured using the tetrazolium blue (NBT) photochemical reduction. The colorimetry was performed at 560 nm, and the 50% inhibition of NBT photochemical reduction was implemented as an enzyme activity unit (U) [21]. All the measurements included three biological replicates. Finally, the SAS (V9.4) was conducted to analyze the variance and Duncan multiple comparisons.

### 2.3. Library Construction and Sequencing

Total RNA was extracted with an RNA Extraction Kit (Tiangen, Beijing, China). The library construction and sequencing were conducted by Novogene company (Novogen, Beijing, China). The libraries were constructed by using the NEBNext^®^ UltraTM RNA Library Prep Kit (Illumina, San Diego, CA, USA) to build the library. The first strand of cDNA was synthesized in the M-MuLV reverse transcriptase system using fragmented mRNA as the template and random oligonucleotides as the primer. Then, the RNA chain was degraded by RNaseH. The second strand of cDNA was synthesized in the DNA polymerase I system using dNTPs as the raw material. Then, the purified double-stranded cDNA underwent end repair, the addition of A tail, and the sequencing connectors were connected. The cDNA of about 250~300 bp was screened with AMPure XP beans and amplified by PCR, and the PCR products were purified with AMPure XP beans again. Finally, the libraries were obtained. After the construction of the library, we used the Qubit2.0 Fluorometer for the preliminary quantification and diluted the library to 1.5 ng/μL. Then, the Agilent 2100 bioanalyzer was employed to detect the insert size of the library. After the insert size met the expectation, qRT-PCR was supposed to accurately quantify the effective concentration of the library (the effective concentration of the library was higher than 2 nM) to ensure that the library was of high quality. After the library passed the inspection, the Illumina HiSeqTM 2000 platform was used to perform next-generation sequencing.

### 2.4. Data Processing and Differential Gene Expression (DEG) Analysis

The raw data obtained from the sequencing were required to undergo quality control. Then, we removed the reads with adaptors and filtered the reads with base mass values less than or equal to 20 and an N ratio greater than 5%. In addition, the HISAT2 (v2.0.5) software was designated to compare clean reads with the teak genome as the reference [22] and use StringTie (1.3.3b) to splice new transcripts [23]. After the transcript was joined, the fragments per kilobase per million mapped reads (FPKM) value of each gene in each sample was calculated using featureCounts (1.5.0-p3) [24]. DEGs were analyzed using DESeq2 (1.16.1) [25], and the *p*-value generated from the original hypothesis test was corrected. *p*_adj_ < 0.05 and |log2 foldchange| > 1 were the judgment standard for significant differences. According to the obtained sequence, the NR and TAIR databases (www.arabidopsis.org) were annotated with blast results, and the E-value was set at 10^−5^. The GO enrichment analysis of DEGs and the statistical KEGG pathway enrichment of DEGs were estimated using clusterProfiler (3.4.4) [26] and with a *p*-value < 0.05, respectively.

### 2.5. Weighted Gene Correlation Network Analysis (WGCNA)

To further explore the interaction between DEGs, we analyzed all the samples using WGCNA. Counts below 60 in all samples were deleted, and the first 90% of genes with variance were screened for WGCNA [27]. The recommended stepwise method was used for analysis, and the minimum number of modular genes was 30. The clustering method identified the highly similar modules, and the digital labels were converted into colors. Then, the highly identical modules were identified via clustering, and the new modules were merged according to the module characteristic genes. MCODE screened the hub gene, and the co-expression network was displayed by Cytoscape (v3.10.1) [28].

### 2.6. qRT-PCR Validation of DEGs

The RNA extraction kit TAKARA MiniBEST Plant RNA Extraction Kit (No.9769), reverse transcription kit PrimeScript RT Master Mix (Perfect Real Time) (No. RR036A), and qRT-PCR kit TAKARA TB Green^®^Premix ExTM Taq (TLi RNaseH plus) (No. RR420A) were purchased from TAKARA company. The RNA extraction and reverse transcription followed the kit instructions. The cDNA product obtained by reverse transcription was standardly diluted to 500 ng for the template. All the qRT-PCR primers (Table 1) were designed by Oligo 7.0 [29], and the qRT-PCR program was carried out according to the manufacturer’s instructions with triplicates. Actin was used as an internal reference gene [30]. Moreover, the relative quantitative results of the genes were calculated by the 2^−ΔΔCt^ method according to the Ct value, and the relative expression difference was analyzed by an amplification efficiency of 100% [31].

### 2.7. Transformation Verification of CBF Gene Function in Teak

In genome-wide identification, we found that two teak *CBFs* (*Tg1g07960* and *Tg06g13830*) have low-temperature responsive cis-acting elements LTR, named *TgCBF2* and *TgCBF4*, respectively. Due to the lack of an established transformation system for teak, we employed the *Arabidopsis* transformation system to verify the functionalities of these two genes. The CDS of *TgCBF2* and *TgCBF4* was cloned according to the gene sequence and inserted into the overexpression vector *pCAMBIA1302* behind the CaMV 35S promoter through *KpnI/SpeI* restriction sites, respectively. Subsequently, the recombinant expression vector was introduced into wild-type *Arabidopsis* (Col-0) via the *Agrobacterium GV3101* strain using the floral dip technique. The positive transformants were selected by MS medium containing 30 μg/mL kanamycin.

The *Arabidopsis* seeds were gathered, their surface was sterilized, and they were placed on a 0.8% agar-solidified MS medium. After a four-day treatment period at 4 °C in the absence of light, the seeds were moved to a long-day photoperiod of 16 h of light and 8 h of darkness at 20 °C. Finally, *Arabidopsis thaliana* was grown at 20 °C, stressed at −4 °C for 12 h, and recovered at 20 °C for 3 days during the rosette stage. The validation of the transformed plants was performed using fluorescence microscopy.

## 3. Results

### 3.1. Teak Exhibits Signs of Damage under Temporary Low Temperatures

**Figure 1:**
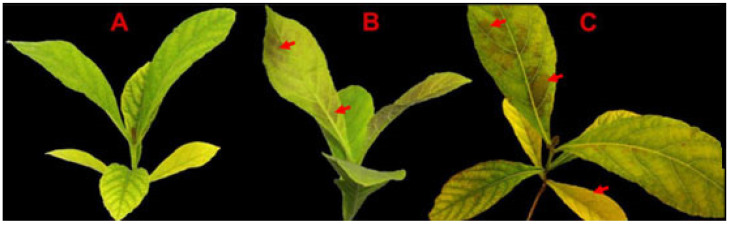
Leaf morphology of teak under cold stress. (**A**): Stress for 0 h; (**B**): Stress for 3 h; (**C**): Stress for 9 h; Arrow: the injured symptoms.

**Figure 2:**
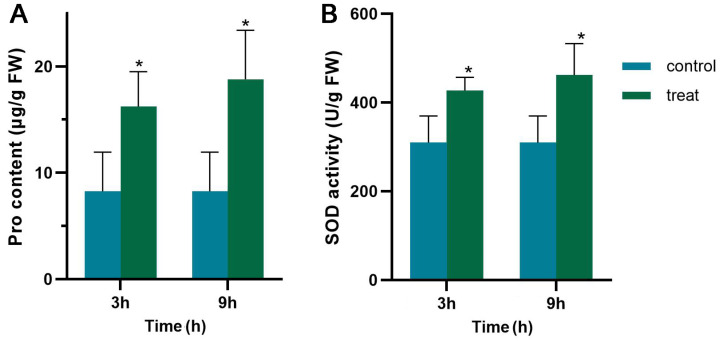
Physiological changes of teak under cold stress. (**A**): SOD content under cold stress; (**B**): Proline content under cold stress. All data are means ± SDs (n = 3). **: *p* < 0.01; *: *p* < 0.05.

- The detrimental effect of cold stress on teak’s leaf morphology is evident from the observed damage at 4 °C (Figure 1). Firstly, the injured symptoms initially occurred at the top of the plant. After 3 h of 4 °C, the top leaves started to present brownish chlorosis spots sporadically, indicating freezing injury (Figure 1B). Subsequently, the top and bottom of the leaves were more injured, and the chlorosis spots increased and enlarged at 4 °C for 9 h (Figure 1C). These results indicate that teak is susceptible to temporary low-temperature stress, with young leaves being particularly vulnerable. These morphological alterations may be connected to secondary metabolites in the leaves.
- Teak showed injury symptoms at low temperatures, as described above, implying physiological and biochemical changes in teak (Figure 2). In this study, it was discovered that the osmotic adjustment index, specifically the free proline content, as well as the oxidative stress index and superoxide dismutase (SOD) content, exhibited a significant increase during short-term low-temperature stress. As a result, during the initial treatment (4 °C for 3 h), the free proline concentration substantially increased from 8.15 μg/g to 16.14 μg/g, representing a significant difference. Moreover, the activity of SOD experienced a noteworthy enhancement from 309.7 U/g to 427.117 U/g. After that, the content of free proline and SOD continuously increased. The content of free proline reached 18.7 μ g/g, and the content of SOD was 461.5 U/g in the second treatment (4 °C for 9 h).

### 3.2. A Total of 1055 Novel Genes Were Identified via Transcriptome Analysis

**Figure 3:**
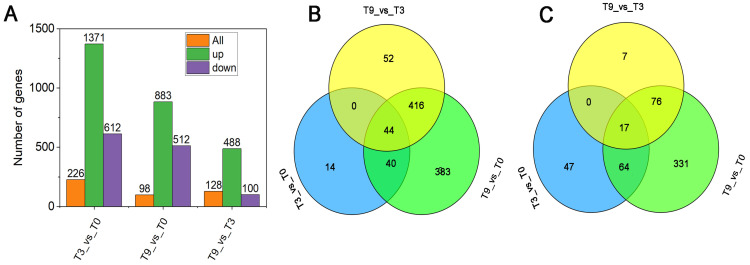
DEGs were identified from 9 samples and venn diagram of DEGs among the three groups. (**A**) DEGs were identified from 9 samples; (**B**,**C**) venn diagram of DEGs among the three groups.T0: Cold stress for 0 h; T3: Cold stress for 3 h; T9: Cold stress for 9 h.

The high-throughput sequencing of nine samples at three stages generated 66.73 G of clean reads, with a minimum of 6.11 G obtained from each sample (Table 2). The reads were uniquely aligned with the teak reference genome (https://datadryad.org/stash/dataset/doi:10.5061/dryad.77b2422, accessed on 15 January 2020) [32] at an efficiency ranging from 84.22% to 89.33%. The reference-guided assembly of mapped reads using the Cufflinks/Cuffmerge pipeline identified 36,901 genes, of which 1055 were novel [33]. Firstly, there were 226 genes between T3 and T9, including 128 down-regulated and 98 up-regulated genes. Secondly, 1371 DEGs were in T9 vs. T0, in which 488 genes were down-regulated, and 883 were up-regulated. Thirdly, we generated 100 down-regulated genes and 512 up-regulated genes in the T3 vs. T0 group (Figure 3A). Among these genes, 44 up-regulated and 17 down-regulated genes were common in the three groups (Figure 3B). qRT-PCR results are shown in Supplementary Materials Figure S1.

### 3.3. Enrichment Characteristics of KEGG and GO of DEGs

**Figure 4:**
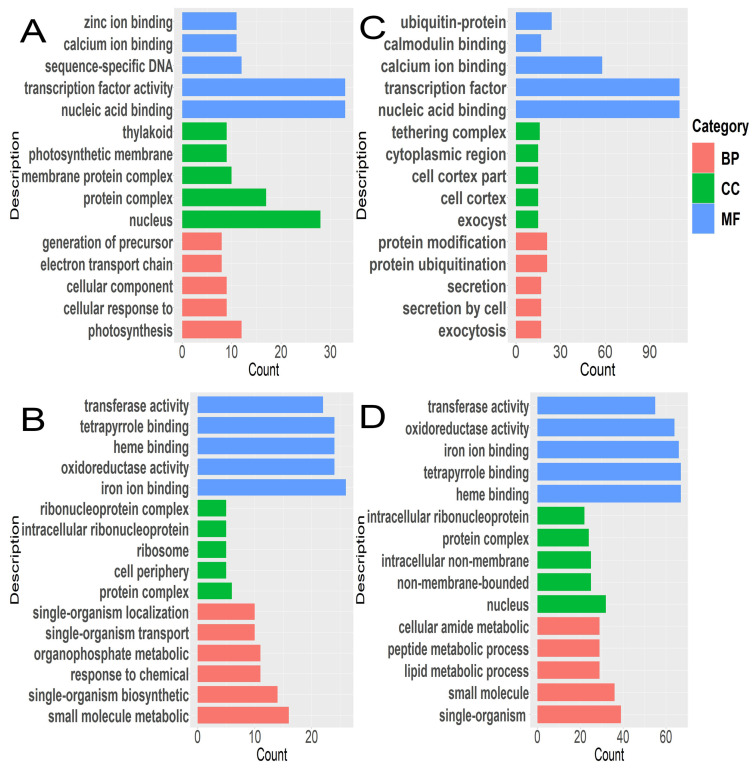
GO analysis of DEGs in teak. (**A**): 3 h up, (**B**): 3 h down; (**C**): 3 h up, (**D**): 3 h down.

A total of 2684 and 13,126 DEGs were assigned GO terms in T3 vs. T0 and T9 vs. T0. After treatment for 3 h at 4 °C, the up-regulated DEGs were primarily enriched in the electronic transmission chain of the photosynthetic system and responded to internal stimuli in the biological process; in terms of cell composition, the enrichment mainly included the nucleus, photosynthetic membrane, and thylakoid and in terms of the molecular function, the enrichment mainly related to transcription factors (*p* < 0.05) (Figure 4A). After 3 h of treatment at 4 °C, the down-regulated DEGs were mainly enriched in the auxin response, hormone response, photosynthesis, and other aspects of the biological process. The cell wall, photosynthetic system, and oxidation–reduction were the most enriched in the cellular composition; this enrichment was mainly related to the oxidation–reduction enzyme activity and transferase activity in the molecular function category (Figure 4B). 

After 9 h of treatment at 4 °C, the up-regulated DEGs were mainly enriched in protein ubiquitination, protein modification, exocytosis, etc., in the biological process; in cell composition, they were mainly enriched in cytoplasm, various perimembranes, etc., and in the molecular function, they were mainly enriched in the calcium-binding protein, ubiquitin transferases, etc. (*p* < 0.05) (Figure 4C). Furthermore, the down-regulated DEGs were mainly concentrated in cell homeostasis, redox homeostasis, protein folding, photosynthetic system, and the biological process; in the cell composition, they were mainly concentrated in the photosynthetic system complex, thylakoid, etc.; in the molecular function, they were mainly concentrated in oxidoreductase activity, transferase activity, etc. (Figure 4D).

**Figure 5:**
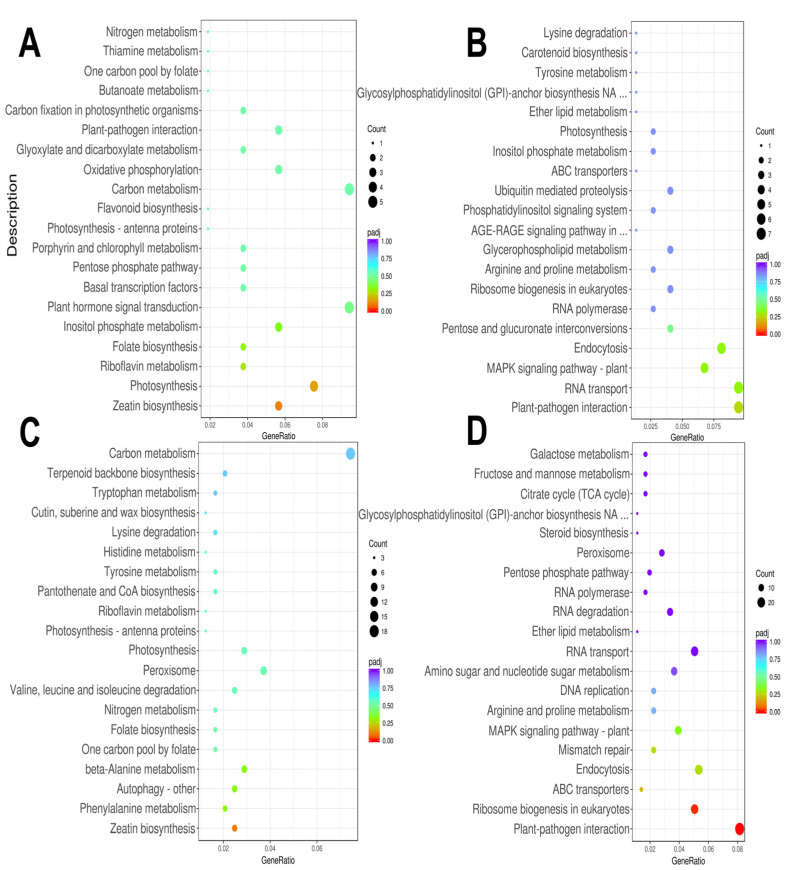
KEGG analysis of DEGs in teak. (**A**): 3 h down, (**B**): 3 h up, (**C**): 9 h down, (**D**): 9 h up.

The KEGG enrichment analysis showed that the most significant down-regulated DEGs were enriched in photosynthesis, plant hormone signal transduction, oxidative phosphorylation, and other pathways (Figure 5A). In contrast, up-regulated DEGs were mainly enriched in the MAPK signal transduction pathway, mutual transformation between pentose and glucuronic acid, ubiquitin-mediated protein decomposition, and so on (Figure 5B). After 9 h of treatment at 4 °C, the DEGs were down-regulated and enriched in autophagy, photosynthesis, and nitrogen metabolism (Figure 5C), while up-regulated DEGs were enriched in plant–pathogen interactions, the MAPK signal transduction pathway, and arginine and proline metabolism (*p* < 0.05) (Figure 5D).

### 3.4. DEGs Involved in Osmoregulation Pathway

**Figure 6:**
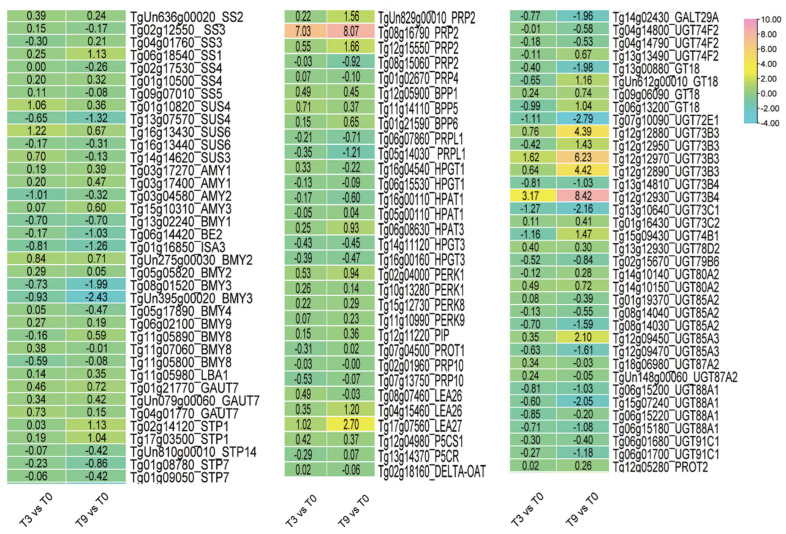
The expression of osmotic regulation genes in teak leaves under cold stress.

In total, 136 genes concerning osmoregulation were detected under low-temperature stress in teak leaves, including 98 genes involved in carbohydrate metabolism and 38 genes relevant to proline and LEA protein. Notable changes were observed in the genes involved in sucrose synthase, UDP-glycosyltransferase, α-Amylase, transporter, and branching enzyme concerning sugar metabolism. The expression of *UGT73B4* (*Tg12g12930*) exhibited a significant 3-fold up-regulation after 3 h at low temperature and a remarkable 8.4-fold up-regulation after 9 h. Nevertheless, the expression of *UGT73C1* (*Tg13g10640*) exhibited significant down-regulation following 3 h of cold stress, with a decrease of 1.27-fold and a further decrease of 2.15-fold after 9 h. Furthermore, following 3 h of treatment at low temperatures, the expression levels of *PRP2* (*Tg08g16790*) and *LEA27* (*Tg17g07560*) exhibited a 7-fold and 1-fold increase, respectively. After 9 h, the expression levels increased by 8-fold and 2.7-fold, respectively (Figure 6).

Proline and soluble sugar are usually indicators of plant cold resistance and the accumulation of the above substances in plants with cold resistance under cold stress is relatively high. We found that only a few proline-synthesis-related genes and *LEAs* changed significantly, which might be related to their role in the osmoregulation of teak (Figure 6).

### 3.5. DEGs Involved in Antioxidant Enzyme Genes under Low-Temperature Stress

**Figure 7:**
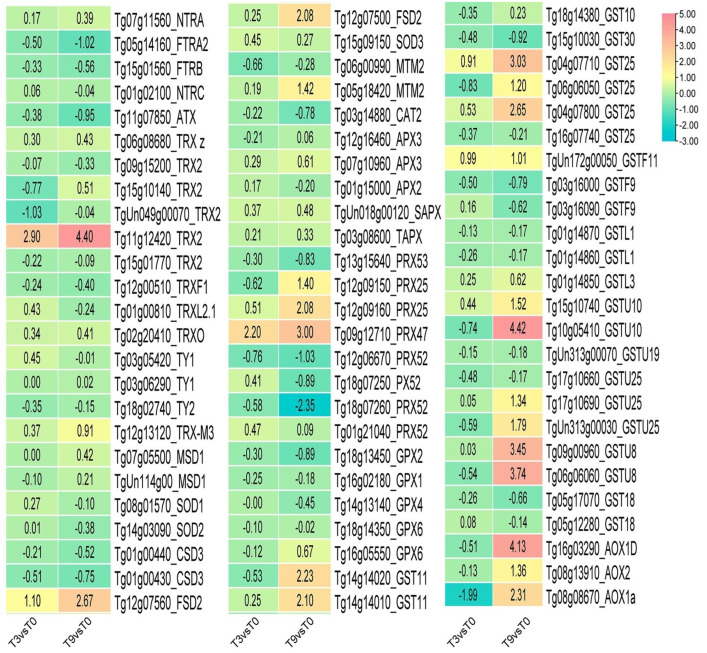
The expression of antioxidant enzyme genes in teak leaves under cold stress.

Under low-temperature stress, 167 antioxidant enzyme genes were detected in teak leaves. The DEGs showed that the expressions of *Trx2* (*TG1G12420*) and *Prx47* (*Tg09g12710*) were most significantly up-regulated after 3 and 9 h of low temperatures. The expression of *PRX52* (*Tg18g07260*) was not considerably down-regulated after 3 h of cold stress but significantly down-regulated after 9 h (Figure 7). 

The results of our research demonstrate that teak leaves experience considerable changes in the expression of antioxidant enzyme genes when exposed to cold stress. Compared to 3 h of cold stress, a significantly higher expression of antioxidant enzyme genes was observed after 9 h. This suggests a prolonged duration of cold stress, increased ROS accumulation, and aggravated cell damage. In teak, detecting nearly all the significant genes responsible for antioxidant enzymes under cold stress highlights the highly conserved regulation of antioxidant genes in evolution.

### 3.6. DEGs Involved in PSI and PSII under Low-Temperature Stress

**Figure 8:**
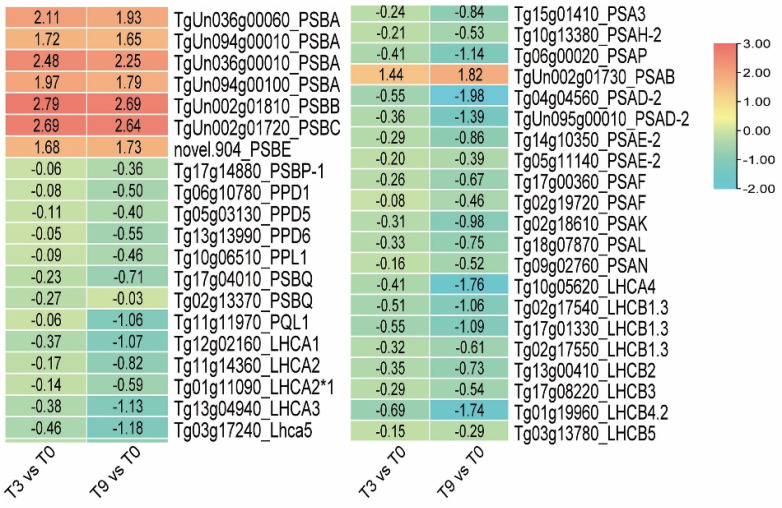
The expression of photosynthetic genes in teak leaves under low-temperature stress.

After 3 h of cold stress, the teak leaves showed chlorosis symptoms, indicating that the photosynthetic system of teak was affected by cold stress. Fifty-six related genes were detected in teak leaves under transient cold stress, involving Photosystem I and Photosystem II core protein, peripheral protein, and antenna protein genes. The analysis of differentially expressed genes revealed significant changes in the expression of several Photosystem II genes in teak after a 3-h exposure to low temperatures. Notably, the expression of *PsbB* (*TgUn002g01810*) was up-regulated by a factor of 2.79, while the expression of *PsbC* (*TgUn002g01720*) was up-regulated by a factor of 2.69, representing the most pronounced changes observed. Following 9 h of cold stress, the expression of these two genes experienced a slight decline, with up-regulation rates remaining at 2.68 and 2.63 times, respectively. The expression of the Photosystem I gene *PsaB* (*TgUn002g01730*) exhibited a 1.4-fold increase following 3 h of cold stress and a 1.8-fold increase after 9 h of cold stress (Figure 8).

According to our research findings, a low-temperature exposure of 3 h resulted in a significant up-regulation of the core protein gene of photosystem I, as well as the up-regulation of the core protein, peripheral protein, and antenna protein genes of photosystem II. These results support the conclusion that PSI exhibits more excellent stability than PSII under low-temperature conditions. Furthermore, it is worth noting that the core protein gene of PSI experienced notable alterations after 3 h, potentially linked to the cold sensitivity of teak.

### 3.7. CBF Expression Pattern under Low-Temperature Stress

**Figure 9:**
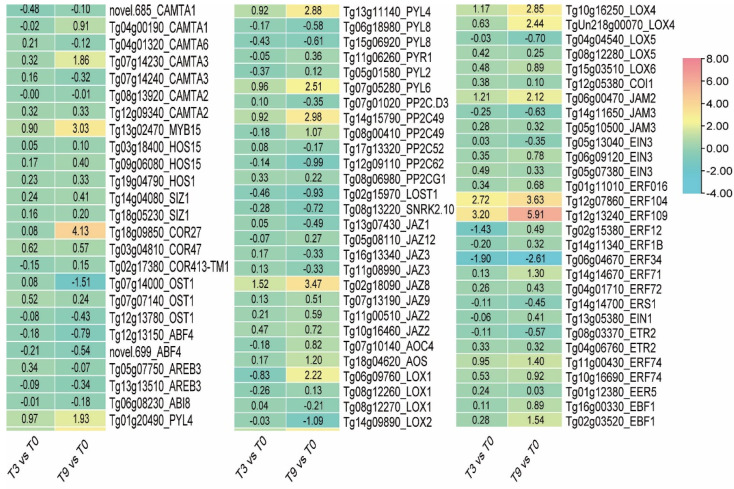
The expression of *CBF*-dependent genes involving cold stress response in teak.

The *ICE1* gene in teak remains relatively stable when exposed to low temperatures, indicating that low temperatures do not significantly impact the *ICE* gene. *CBF2* (*TgUn004g00050*) and *CBF4* (*Tg06g13830*) exhibited up-regulation following 3 h of exposure to low temperatures, although the increase in the *CBF2* expression was relatively modest. The up-regulation of the *CBF4* expression was significant, with a 3.66-fold increase. The expression of *CBF2* showed minimal change after 9 h, whereas the expression of *CBF4* was significantly up-regulated by 7.2 times (Figure 9). These results demonstrate that low temperature led to the induction of *CBF2* and *CBF4* genes in teak, resulting in significant changes in the expression of *CBF4*.

**Figure 10:**
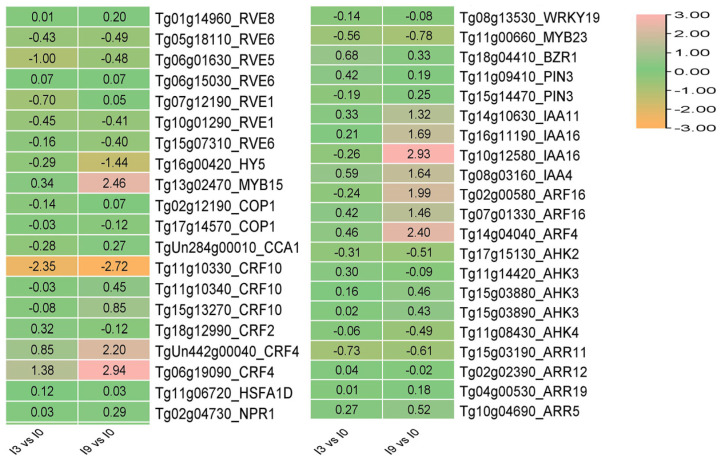
The expression of *CBF*-independent genes involving cold stress response in teak.

In terms of hormonal regulation pathways, after 3 h of exposure to low temperatures, the expressions of *JAZ8* (*Tg02g18090*), *LOX4* (*Tg10g16250*), and *JAM2* (*Tg06g00470*), which are essential genes of the jasmonic acid pathway in teak, were significantly up-regulated by 1.52, 1.17, and 1.21 times, respectively. Nevertheless, the expression levels of *ERF34* (*Tg06g04670*) and *ERF12* (*Tg02g15380*), which are crucial genes in the ethylene pathway, exhibited significant down-regulation. Following 9 h of exposure to cold stress, the expression levels of *JAZ8* (*Tg02g18090*), *LOX4* (*Tg10g16250*), *JAM2* (*Tg06g00470*), *ERF104* (*Tg12g07860*), and *ERF109* (*Tg12g13240*) exhibited significant up-regulation by 3.47-, 2.85-, 2.12-, 3.63-, and 5.91-fold, respectively, while *ERF34* (*Tg06g04670*) showed a decrease of 2.61-fold. Following a period of 9 h of exposure to cold stress, the expressions of the genes involved *AOS* (*Tg18g04620*), *PP2C49* (*Tg14g15790*, *Tg08g00410*), *PYL4* (*Tg01g20490*, *Tg13g11140*), *PYL6* (*Tg07g05280*), and *EBF1* (*Tg02g03520*) were significantly up-regulated by 1.20, 2.98, 1.07, 1.93, 2.88, 2.51, and 1.54 times, respectively (Figure 9). Additionally, the expression levels of *HOS1*, *EGR2*, *ABI1*, *BTF3*, and *MYBS*, which are the genes that regulate the *CBF* pathway, exhibited minimal alteration in teak under cold stress. However, *OST1* (*Tg07g14000*) displayed minimal change after 3 h of cold stress but experienced a significant down-regulation after 9 h. After 3 h of exposure to low-temperature stress, no notable alterations were observed in the *CAMTA* gene. Subsequently, after 9 h of stress, the expression of *CAMTA3* (*Tg07g14230*) exhibited a significant up-regulation of 1.86-fold (Figure 10). The findings of this study support the conclusions of previous research in that ABA, ET, SA, JA, and GA play a role in the response of plants to cold stress either independently or through an interaction. Simultaneously, the situation above also suggests that the ABA, JA, and ethylene pathways assume a pivotal function in teak’s response to low-temperature stress.

### 3.8. The Expression of CBF-Independent Genes HY5 and REV5 in Teak Changed Dramatically in Response to Cold Stress

In addition to *CBF* genes, there were other *CBF*-independent pathway genes involved in the plant response to cold stress, such as *HY5*, *REV4*, *REV8*, *CCA1*, and so on. After 3 h of low-temperature stress, the expression of *CRF4* (*Tg06g19090*), an essential gene in the cytokinin pathway of teak, was significantly increased, while the expression of *REV5* (*Tg06g01630*) was significantly decreased by 1.0 times, respectively. After 9 h of low-temperature stress, the expression of *HY5* (*Tg16g00420*) decreased significantly by −1.44 times (Figure 10). The expression of the *HY5* and *REV5* genes in teak changed significantly, indicating that they may play an essential role in teak’s response to low-temperature stress.

### 3.9. Calcium Signal Transduction in Teak Cells under Low-Temperature Stress

Cold sensing is initial for plant genes reacting to cold stress. In rice, the perception and transmission of cold stress signals are related to G protein regulators *COLD1* (chilling tolerance divergence 1), *RGA1* (rice G-protein α subunit 1), and *OSCIPK7* (CBL-interacting protein kinase 7) [34,35]. However, the *COLD1* homologous gene was not detected in teak, and the expression of *RGA1* homologous genes (*Tg05g00750*) and *CIPK7* (*Tg04g06110*) in teak rarely changed after 3 or 9 h of stress. It is probable that the cold sensor of teak was different from that of rice, or this finding might be related to the sampling period.

**Figure 11:**
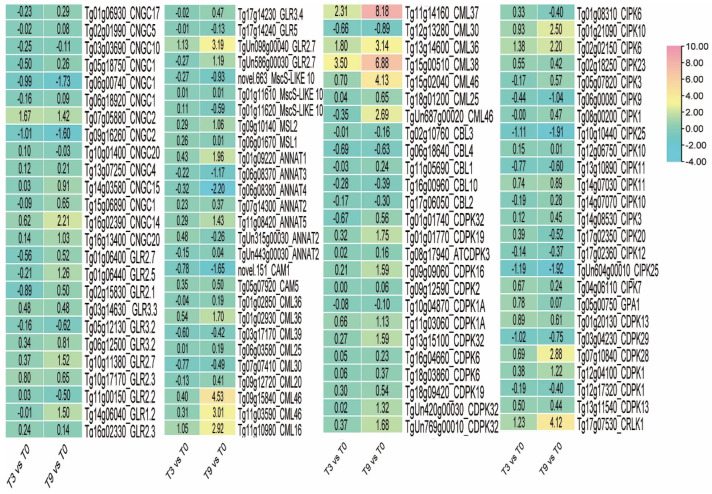
The expression of Ca^2+^ signal transduction pathway genes in teak.

In addition, Ca^2+^ is a vital messenger in plant cells. After 3 h of cold stress, *CNGC2* (*Tg07g05880*) and *GLR2.7* (*TgUn098g00040*) had the largest up-regulated amplitude, by 1.67 and 1.13 times, respectively, and *CNGC2* (*Tg09g16260*) had the largest down-regulation amplitude, by −1.01 times. After 9 h of cold stress, the up-regulation of the expression of *GLR2.7* (*TgUn098g00040*) was the largest, by 3.19 times, while the down-regulation of *CNGC1* (*Tg06g00740*) was the largest, by −1.73 times. The above results show that most of the protein genes involved in the Ca^2+^ influx are detectable, and the gene expression was significantly up-regulated or down-regulated, indicating that Ca^2+^ influx occurred in teak cells under cold stress. Furthermore, it was observed that there were no notable alterations in the *CBL* gene, thus suggesting that the calcium signaling and interpretation in teak differ from those in other plant species (Figure 11).

### 3.10. WGCNA and Hub Genes of DEGs Identified Three CBF-Dependent and CBF-Independent Regulatory Networks

**Figure 12:**
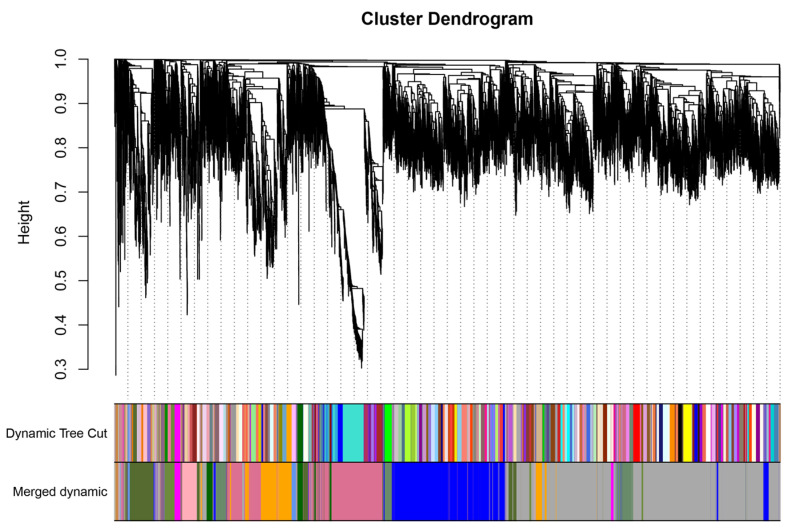
The module identification from WGCNA Networks.

WGCNA identified gene clusters that co-expressed under cold stress in teak. In total, 49 7726 DEGs were clustered into 14 modules; each module contained 45 to 1640 genes (Figure 12). Among these modules, the orange module was highly correlated with the *CBF2* pathway, the palevioletred module was highly correlated with the *CBF4* pathway, and the cornflower blue module was relatively associated with the *HY5* pathway. 

**Figure 13:**
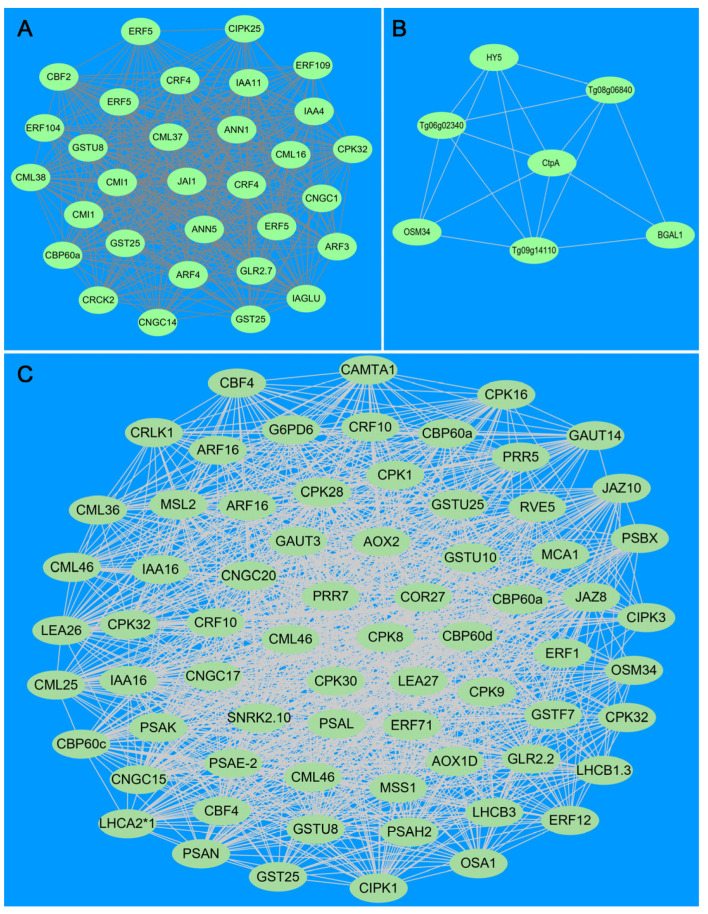
Co-expression networks of Teak hub genes under cold stress. (**A**): Co-expression network of Teak hub genes in the orange module; (**B**): Co-expression network of Teak hub genes in the palevioletred module; (**C**): Co-expression network of Teak hub genes in the cornflowerblue module.

In the orange module, there were 937 genes, and the sub-network of *CBF2* included 576 genes, where 311 genes were annotated and 31 hub genes were screened out to form a co-expression network (Figure 13A). These hub genes include four membrane channel protein genes (*ANN*, *CNGC*) and 10 calcium-binding protein genes (*CML*, *CPK*, *GLR*), which mediate calcium influx. Only *CBF2* was included in the *CBF* pathway. In addition, the network also comprises three antioxidant genes (*GST*) and 13 hormone-related genes (*ARF*, *IAA*, *CRF*, *ERF*, *JAI1*). The co-expression network showed that the *CBF2* of teak was induced by a low temperature and interacted with many hormones.

In the palevioletred module, there were 1640 genes, including 852 annotated genes and 69 hub genes; their co-expression network is outlined in Figure 13B. These hub genes include six channel protein genes (*CNGC*, *GLR*, *MCA*, *MSL*) and 13 calcium-binding protein genes (*CPK*, *CML*, *CBP*) that mediate calcium influx, including *CBF* pathway genes, including the core gene *CBF4*, its upstream regulator *CAMTA1*, and its downstream gene *COR27*. The co-expression network also generated five osmotic adjustment genes (*LEA*, *OSM*, *G6PD6*), 10 antioxidant genes (*AOX1a*, *GAUT*, *GST*), six photosystem I genes, and two photosystem II genes. Five hormones participated in the co-expression network, namely four auxin response genes (*ARF*, *IAA*), two cytokinin response genes (*CRF*), three ethylene response genes (*ERF*), two jasmonate-related genes (*JAZ*), and an abscisic acid pathway gene (*SNRK*). The co-expression network showed that the *CBF4* of teak was induced by a low temperature and regulated *COR27* and was held by *CAMTA1* upstream.

In the cornflower blue module, the sub-network of *CBF*, which is independent of the pathway gene *HY5*, included seven genes, of which only four were annotated (Figure 13C). This suggested that the *HY5* gene directly interacted with the osmoregulation gene *OSM34,* and *HY5* of teak never interacted with *CBF*, but it can participate in cold stress response.

### 3.11. Morphologies of OE-TgCBF2 and OE-TgCBF4 under Low-Temperature Treatment

**Figure 14:**
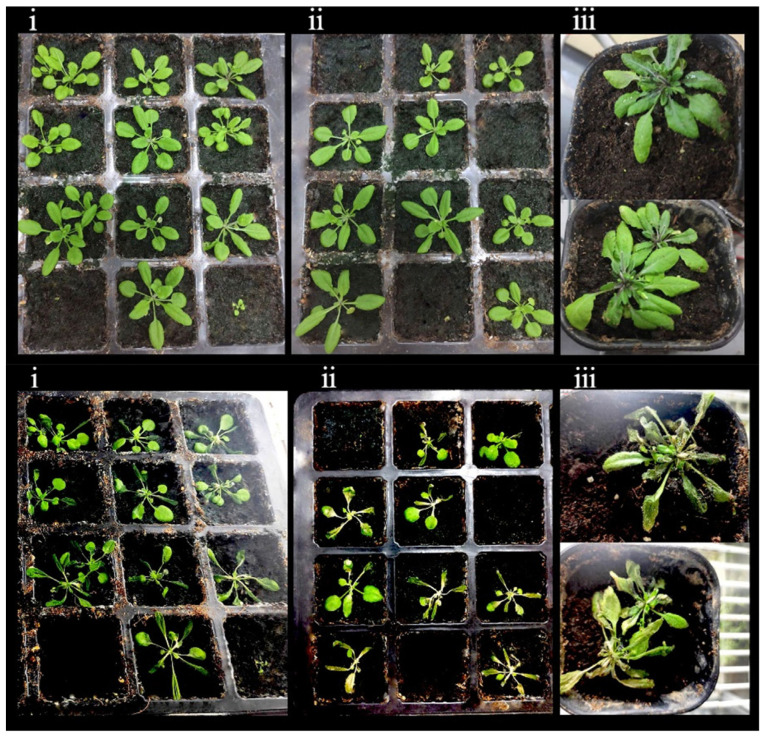
The morphology of transformants (**top**) and wild type (**bottom**) before low-temperature treatment. (**i**):*OE-TgCBF2*; (**ii**): *OE-TgCBF4*; (**iii**): Control.

**Figure 15:**
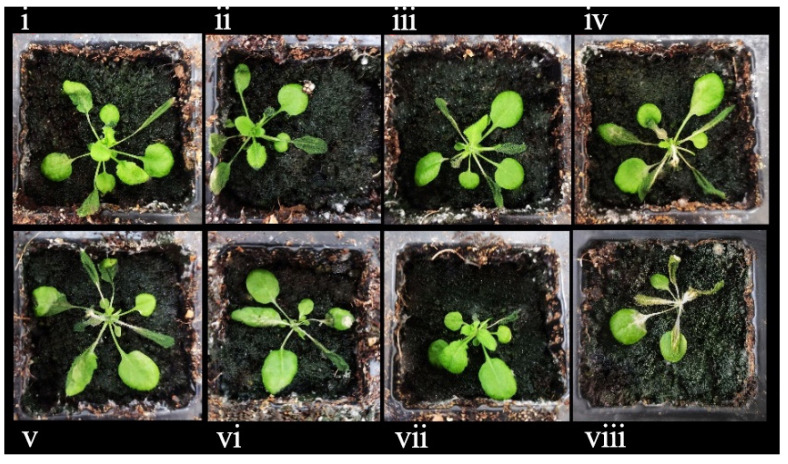
The morphology of transformants and wild type after 3 days of recovery from low-temperature stress. (**i**–**iv**): *OE-TgCBF2*; (**v**–**viii**): *OE-TgCBF4*.

**Figure 16:**
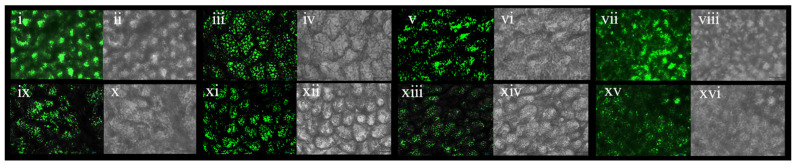
The fluorescence verification of transgenic Arabidopsis. (**i**–**viii**): *OE-TgCBF2* transformants fluorescence verification. (**ix**–**xvi**): *OE-TgCBF4* transformants fluorescence verification. (**i**,**iii**,**v**,**vii**,**ix**,**xi**,**xiii**,**xv**): Fluorescence image; (**ii**,**iv**,**vi**,**viii**,**x**,**xii**,**xiv**,**xvi**): Bright-field image.

The cold resistance test was carried out in the rosette stage of *A.thaliana* (Figure 14). The survival rate of *OE-TgCBF2* was about 70%, and that of *OE-TgCBF4* was about 30% (Figure 14). Eight representatives of *Arabidopsis*, which performed well in the cold resistance test, were observed by fluorescence, and all the plants were detected with fluorescence signals, indicating the normal expression of the genes (Figure 15 and Figure 16). These results show that the survival rate of transformants was significantly higher than that of wild-type under low-temperature stress. Also, both *TgCBF2* and *TgCBF4* genes boosted the cold resistance, and *OE-TgCBF2* displayed better cold resistance.

## 4. Discussion

Given the sensitivity of the tropical plant teak to cold stress, it is imperative to thoroughly investigate the molecular mechanisms underlying its response to cold stress to facilitate a genetic improvement through breeding. This study elucidated that the induction of *CBF2* and *CBF4* in the *CBF*-dependent teak pathway occurred due to a rapid drop in temperature, thereby contributing to its cold resistance mechanism. Moreover, it was observed that HY5 was also activated in response to a swift drop in temperature. This discovery demonstrates the existence of both *CBF*-dependent and *CBF*-independent mechanisms for teak’s resistance to cold stress.

### 4.1. Teak CBFs and Their Regulation Exhibit Conservation and Differentiation

Previous research has demonstrated the pivotal role played by *CBFs* in plant resistance to cold stress, with the signal pathway of *CBF* dependence being particularly well-defined. The expression of *CBF1*, *CBF2*, and *CBF3* among the four *CBFs* in Arabidopsis thaliana was induced by the low temperature, but among the three *CBFs* of tomato, only *CBF1* was induced by the low temperature [36]. According to our findings, teak *CBF1* and *CBF3* do not exhibit low-temperature induction, whereas *CBF2* and *CBF4* are induced by low temperatures. Functional redundancy and differentiation among *CBF* members in various plants may represent the distinct strategies different plants employ to mitigate multiple environmental stresses. Regarding *CBF* regulation, it is worth noting that while the *ICE1* gene was identified in teak, its expression level was notably low. *ICE1* is constitutive and enhances the expression of *CBF3* under low temperatures; whether the absence of the expression of *CBF3* is due to the lack of the induction of *ICE1* or other reasons requires further discussion. The role of the regulatory factor *HOS1* is to degrade *ICE1*. The expression of *HOS1* (*Tg19g04790*) in teak was slightly up-regulated under cold stress, but its expression was deficient. A previous study suggested that *CAMTA3* regulates the expression of *CBF2* and also regulates the expression of *CBF1* when the temperature decreases rapidly [11]. Our study showed that the expression of *CAMTA3* (*Tg07g14230*) had little change after 3 h of cold stress, and then the expression of *CAMTA3* (*Tg07g14230*) was significantly up-regulated after 9 h of cold stress. Moreover, the expression of *CBF2* was continuously up-regulated under cold stress, but the expression of the *CBF1* gene was not affected. The *CAMTA3* barely participated in the regulation of *CBF1* when the temperature decreased rapidly from 20 °C to 4 °C, indicating different cold regulation mechanisms in teak. The results show the presence of diverse regulatory pathways involving *ICE*-*CBF*-*COR*.

### 4.2. The Signal Transduction of Cold Stress in Teak Also Exhibits Conservation and Diversity

The transduction of the cold stress signal is conservative in plants and differentiated in different plants. For instance, rice’s *COLD1*, *RGA1*, and *CIPK7* proteins were related to the perception of the cold stress signal [34,35]. However, the *COLD1* homologous genes were not discovered in teak. Although *RGA1* and *CIPK7* genes could be found in teak, the expression of these two genes exhibited no significant difference, whether after 3 h or 9 h of low-temperature stress. It is thought that they perhaps play a role in the early stage of cold stress, and it is impossible to determine whether they participate in the perception of a cold signal according to the data of 3 h of cold stress. Additionally, the cold stress perception of teak could perhaps differ from other plants and may also be related to the sampling period. The gene expression of calcium channel proteins *GCNC*, *GLR*, *MSL*, *ANN*, and *MCA* that mediate the Ca^2+^ influx changed significantly after 3 h of cold stress, and the gene expression of the cytoplasmic calcium-binding proteins *CaM*, *CML*, *CDPK*, and *CIPK* also changed significantly, which fully shows that Ca^2+^ influx caused the change in the intracellular Ca^2+^ concentration. However, it is noteworthy that the expression of *CBL* does not change significantly under cold stress. After binding with Ca^2+^, CIPK phosphorylates CBL, and CBL activates the downstream gene cascade response [37]. The change in *CBL* under cold stress was insignificant, which reflected that the signal transduction pathway of teak under cold stress was different from that of other plants. Under cold stress, there were changes in the expression of multiple members of *OST1* and *PP2C* in the ABA pathway of teak, but downstream *ABI3* and *ABI5* were not detected. Although *ABI8* was highly expressed, the change was not significant. The *MYC2* gene in the JA pathway of teak was significantly up-regulated after 3 h and 9 h of cold stress, but *COI1* and *JAZ* did not change much under cold stress. Ethylene seems to depend on species to participate in the cold stress response, which positively regulates the cold tolerance of apples and oranges and negatively regulates the cold tolerance of *Medicago truncatula* and *A. thaliana* [38,39]. The expression of several ethylene pathway genes in teak changed significantly, but how they work under the cold stress of teak deserves further discussion.

### 4.3. The CBF-Independent Pathway Also Influences the Resistance of Teak against Cold Stress

A low temperature positively regulates the expression of *HY5* at transcription and post-translation levels [40,41,42], but the expression of *HY5* (*Tg16g00420*) in teak was down-regulated under cold stress, and the gene expression was down-regulated after 3 h of cold stress. Still, it did not reach a significant level. After 9 h of cold stress, the expression was significantly down-regulated by −1.44 times. In addition, the expression of *RVE5* was significantly down-regulated under cold stress. The above situation not only reflects the complexity of the response of teak to cold stress. Under cold stress, the expression of *HSFA1* was slightly up-regulated, while *NPR1* and *COP1* were slightly down-regulated, and the changes were insignificant. Perhaps independent *CBF* pathways such as *HY5* and *RVE5* played an essential role in the response of teak to cold stress.

## 5. Conclusions

Teak, a tropical plant, seldom encounters low temperatures throughout its growth and development. Despite the lack of clarity regarding the mechanism of its resistance to cold stress, some genes, namely *CBF* and *HY5*, exhibit resistance to cold stress. Furthermore, the process of genetic transformation demonstrates the capacity of teak *CBF2* and *CBF4* to enhance the survival rate of teak seedlings when subjected to sudden temperature decreases. Consequently, it can be excavated and utilized to improve cold resistance, thereby contributing to the genetic enhancement of teak. The conservative nature of *CBF* genes also implies that introducing *CBF* genes from cold-resistant plants could improve the cold resistance of teak. Additional investigations into the regulatory mechanism of teak *CBF* in response to cold stress will facilitate the development of cold-tolerant teak using gene editing techniques.

## Figures and Tables

**Table 1:** Genes and primers used in qRT-PCR.

Gene ID	Forword Primers	Reverse Primers	TAIR Description
*Tg01g18270*	AATCTGACGATTCGCAACCC	CGAGCAGTACAATCCTCTCCC	*NDR1/HIN1-like 2*
*Tg07g05280*	AGAGCTGCCACGTCATCCTTG	CCACCACGCTGAAACTCGTC	*RCAR9*, regulatory components of ABA receptor 9
*Tg09g03150*	AGACTTCTAGATAAAGCTCGT	TTTCTCTATCCGCCACCGTA	*SAUR30*, small auxin upregulated rna 30
*Tg09g12620*	TCACTTTCCACAGAAGGCATC	GAACACGACATCGCTCCAC	AATP1, AAA-ATPase 1
*Tg16g05170*	TGCTGGTCTACCTATTGACAGT	CCAAATCGGAGAACTTCACCAC	*BRH1*, brassinosteroid-responsive RING-H2
*TgUn002g01760*	TTTGACTGATCCTGCCCCTG	TGTTCCTCACCAACGATCCGA	*ATPB*, ATP synthase subunit beta
*TgUn195g00060*	TGGTATATCTCTTCCGGTGT	TCATCGCCATCAAAATCTCC	*CBP60a*, Calmodulin-binding protein 60a
*Tg06g13830*	CGTCATCGGATCCTAAGGACA	CCTCCTCATCCATAAAGCACAC	*CBF4*
*Tg16g06080*	GAGCTACGTGAGCCAACCCAA	GCAACCGCCATACAGAGTCC	*DDF1*, Dwarf and delayed flowering 1
*TgUn466g00010*	AGGTCAGCAACAATTACACGG	ATTTCTCGGCAATTCCAGGTT	*WRKY6*
*Tg03g16060*	TCCAGGCTCAATATCCACCAC	GCCACCCATTTTCCCCAGT	*WIND1*, wound induced dedifferentiation 1
*Tg09g10240*	TCCGCCAGACTCTTTACTCCAC	CGAACCATTGCGACATCAGCAG	*DIC2*, dicarboxylate carrier 2
*Tg03g10220*	GCCTTTGGAGCTTCAGCAAC	CCCGAGAGAGCAAAACACGAT	*SUT1*, SUCROSE TRANSPORTER 1
*Tg13g10760*	TTCTGCCAAGACAAACACCAG	ATTCCGCCATCTATTTCACCAC	*FER*, FERONIA
*Tg05g13790*	GTTGAACATGCTGCTACTCAC	TAGCTTTGCCCAAAATTCCAC	*bZIP 23*
*Tg06g00470*	TGTTCCCAAGATTTTCGGACA	TGTAGCGCCATGAAAACCA	*JAM2*, Jasmonate Associated MYC2 LIKE 2
*Tg01g19010*	TGAATTTAGCCTGCAGCCAA	TCAAATCCCCGTACATCCAC	*LHW*, LONESOME HIGHWAY
*Tg18g02580*	TTGAGACCTTCAACGTGCC	ATAATCAGTGAGATCCCGACCA	*Actin*

**Table 2:** Assembly statistics for the teak transcriptome.

Sample	Raw_Reads	Clean_Reads	Clean_Bases	Q30 (%)	Total_Map	Splice Reads(%)	GC (%)
T0_1	42,258,920	40,712,072	6.11 G	93.4	36,128,928 (88.74%)	12,002,426 (29.48%)	44.82
T0_2	45,101,940	44,351,094	6.65 G	93.32	39,455,647 (88.96%)	13,123,149 (29.59%)	44.53
T0_3	52,698,330	50,794,162	7.62 G	93.16	45,374,885 (89.33%)	15,642,780 (30.8%)	44.90
T3_1	61,704,656	59,371,024	8.91 G	93.37	52,288,275 (88.07%)	17,879,136 (30.11%)	44.29
T3_2	59,390,990	57,148,694	8.57 G	93.22	50,258,279 (87.94%)	15,592,721 (27.28%)	43.93
T3_3	47,792,662	45,960,592	6.89 G	93.04	40,063,421 (87.17%)	13,199,390 (28.72%)	43.72
T9_1	53,340,150	51,332,880	7.70 G	93.24	44,434,695 (86.56%)	13,681,417 (26.65%)	43.77
T9_2	55,819,212	53,679,450	8.05 G	93.19	46,979,435 (87.52%)	14,590,252 (27.18%)	44.13
T9_3	43,179,072	41,514,078	6.23 G	93.41	34,961,459 (84.22%)	10,024,613 (24.15%)	44.35

## Data Availability

Data are contained within the article and Supplementary Materials.

## Supplementary Materials

The following supporting information can be downloaded at: https://www.mdpi.com/article/10.3390/genes14112098/s1, Figure S1: Validation of DEGs by qPCR.
