# Supplementary figures and images for: Transcriptomic Analysis Reveals CBF-Dependent and CBF-Independent Pathways under Low-Temperature Stress in Teak (Tectona grandis)
Source: Genes (Basel). 2023 Nov 18;14(11):2098. doi: 10.3390/genes14112098 (PMC10670985; doi:10.3390/genes14112098)

## Supplementary Materials

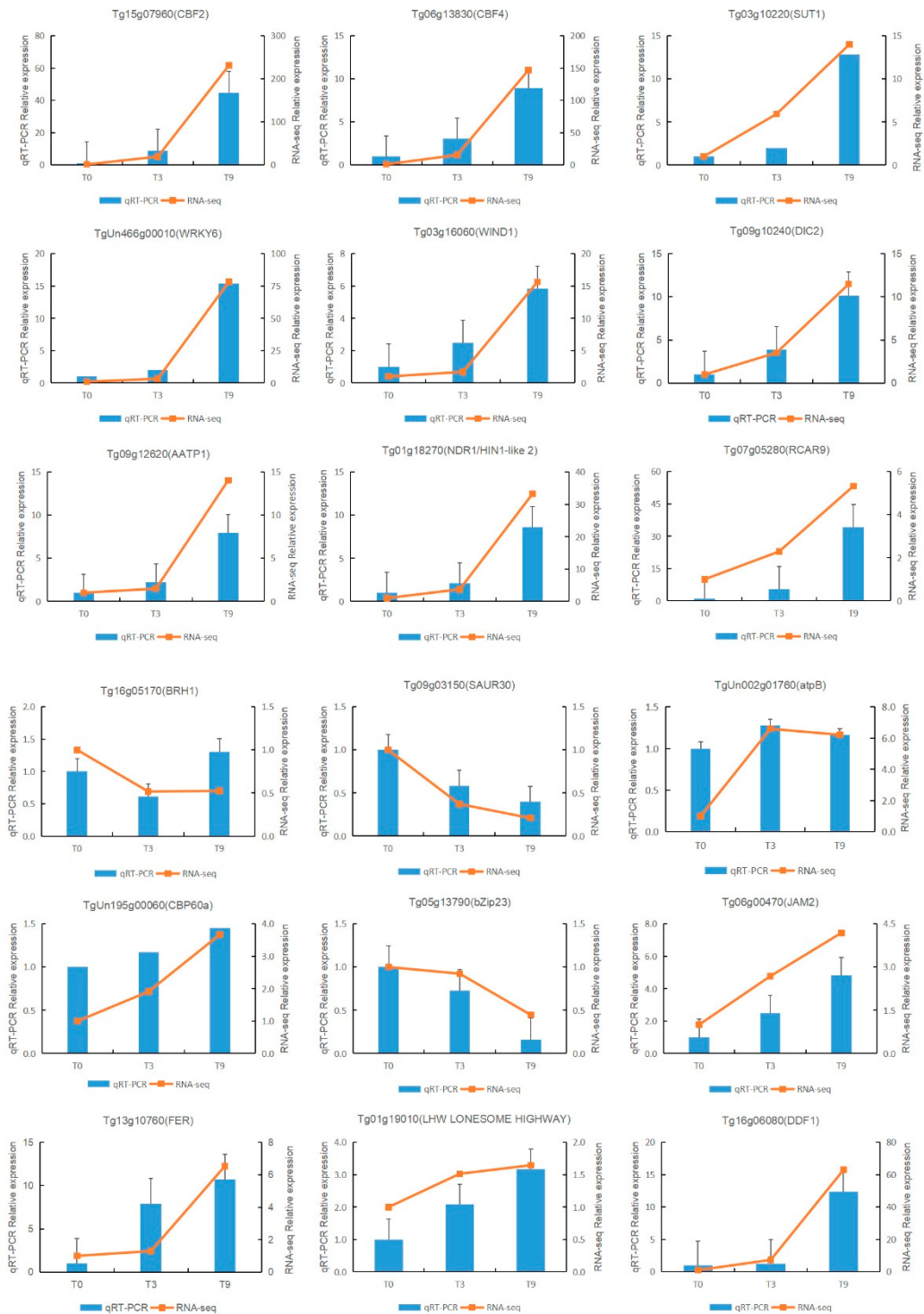

Figure S1. Validation of DEGs by qPCR.

Supplement: Supplementary file 1 [file genes-14-02098-s001.zip › genes-2685896-supplementary.pdf]
